# Genome-Wide Study of *Hsp90* Gene Family in Cabbage (*Brassica oleracea* var. *capitata* L.) and Their Imperative Roles in Response to Cold Stress

**DOI:** 10.3389/fpls.2022.908511

**Published:** 2022-06-22

**Authors:** Shoukat Sajad, Shuhan Jiang, Muhammad Anwar, Qian Dai, Yuxia Luo, Muhammad A. Hassan, Charles Tetteh, Jianghua Song

**Affiliations:** ^1^College of Horticulture, Vegetable Genetics and Breeding Laboratory, Anhui Agricultural University, Hefei, China; ^2^Guangdong Technology Research Center for Marine Algal Bioengineering, Guangdong Key Laboratory of Plant Epigenetics, College of Life Sciences and Oceanography, Shenzhen University, Shenzhen, China; ^3^School of Agronomy, Anhui Agricultural University, Hefei, China; ^4^Department of Plant Pathology, College of Plant Protection, Anhui Agricultural University, Hefei, China

**Keywords:** cabbage, genome wide, *Hsp90*, expression pattern, phylogenetic analysis

## Abstract

Heat shock protein 90 (Hsp90) plays an important role in plant developmental regulation and defensive reactions. Several plant species have been examined for the *Hsp90* family gene. However, the *Hsp90* gene family in cabbage has not been well investigated to date. In this study, we have been discovered 12 *BoHsp90* genes in cabbage (*Brassica oleracea* var. *capitata* L.). These *B. oleracea Hsp90* genes were classified into five groups based on phylogenetic analysis. Among the five groups, group one contains five *Hsp90* genes, including *BoHsp90-1*, *BoHsp90-2*, *BoHsp90-6*, *BoHsp90-10*, and *BoHsp90-12*. Group two contains three *Hsp90* genes, including *BoHsp90-3*, *BoHsp90-4*, and *BoHsp90*. Group three only includes one *Hsp90* gene, including *BoHsp90-9*. Group four were consisting of three *Hsp90* genes including *BoHsp90-5*, *BoHsp90-7*, and *BoHsp90-8*, and there is no *Hsp90* gene from *B. oleracea* in the fifth group. Synteny analysis showed that a total of 12 *BoHsp90* genes have a collinearity relationship with 5 *Arabidopsis* genes and 10 *Brassica rapa* genes. The promoter evaluation revealed that the promoters of *B. oleracea Hsp90* genes included environmental stress-related and hormone-responsive *cis-elements*. RNA-seq data analysis indicates that tissue-specific expression of *BoHsp90-9* and *BoHsp90-5* were highly expressed in stems, leaves, silique, and flowers. Furthermore, the expression pattern of *B. oleracea BoHsp90* exhibited that *BoHsp90-2, BoHsp90-3, BoHsp90-7, BoHsp90-9, BoHsp90-10, and BoHsp90-11* were induced under cold stress, which indicates these *Hsp90* genes perform a vital role in cold acclimation and supports in the continual of normal growth and development process. The cabbage *Hsp90* gene family was found to be differentially expressed in response to cold stress, suggesting that these genes play an important role in cabbage growth and development under cold conditions.

## Introduction

Crop plants during their growth cycle often confronted with various biotic and abiotic stresses (Food and Agriculture Organization [FAO], 2020). Recent climatic changes continuously trigger extreme temperature events and disrupt optimal plant growth (IPCC, 2021; Masson-Delmotte et al., 2021). Increased global warming instigated the risk of cold damage to crop plants (Gu et al., 2008; Moreno and Orellana, 2011; Hassan et al., 2021). Plants contain regulatory mechanisms that enable them to withstand harsh environmental conditions, called acclimatization (Liu et al., 2019). To acclimatize the extreme temperature (low and high) conditions, plants exhibit a number of protein expressions either to promote biosynthesis of new proteins or to protect existing proteins; cold acclimation process carried through expressions of highly conserved stress proteins called heat shock proteins (Hsps; Queitsch et al., 2002). Various types of Hsps are particularly found in all plant species (Xiong and Ishitani, 2006). Based on molecular mass, Hsps were categorized into five groups, i.e., the sHsp family, the chaperonin (Hsp60/GroEL) family, the 70-kDa heat shock protein (Hsp70/DnaK) family, the Hsp90 family, and the Hsp100/ClpB family (Pratt and Toft, 2003; Wang et al., 2004; Al-Whaibi, 2011).

The Hsp90 protein family is a renowned and extremely conserved class of molecular chaperones in eukaryotic cytoplasm (Chen et al., 2006).” For instance, in *Arabidopsis*, there are 7 Hsp90 proteins, of which AtHsp90-1, AtHsp90-2, AtHsp90-3 and AtHsp90-4 are present in the cytoplasm, while AtHsp90-5, AtHsp90-6 and AtHsp90-7 are present in the mitochondria, chloroplast, and endoplasmic reticulum, respectively (Krishna and Gloor, 2001; Yamada et al., 2007). It is a part of the ATPase superfamily (GHKL: gyrase, histidine kinase, and MutL; Dutta and Inouye, 2000); It functions as an ATP-regulated dimer and chaperone protein with three highly conserved domains: a 25 kDa C-terminal substrate-binding domain, a 12 kDa intermediate ATP-binding domain, and a 12 kDa N-terminal ATP-binding domain (Prodromou et al., 1997; Pearl and Prodromou, 2000).” The protein channel that binds to ATP in Hsp90 is frequently closed (Sung et al., 2016) which results in the poor activity of N-terminal ATPase (Raman and Suguna, 2015). The cytoplasm of eukaryotic cells contains the intermediate and N-terminal domains of Hsp90; the charged portions are varied in length between species (Pearl and Prodromou, 2000). It is commonly known that a protein’s function is determined by its ability to fold into a three-dimensional structure. The Hsp90 proteins together with other molecular chaperones provide a course that facilitates protein folding (Wang et al., 2004). The *Hsp90* gene family suppress protein aggregation and enable the ubiquitination of inactive proteins (Picard, 2002); further, it belongs to a group of molecular chaperones that are involved in the creation of the spatial structure of kinase substrates, DNA repair and substrate activation, early stress signaling, and transcription factor spatial structure maintenance (Jackson et al., 2004; Lachowiec et al., 2015).” The expression of stressor *Hsp90* gene is upregulated when plants are stressed; it interacts and repairs the deformed proteins with support of non-proteinaceous compounds (Mukhopadhyay et al., 2003).

This protein (Hsp90) has been found in a number of plant species such as 7 in *Arabidopsis* (Krishna and Gloor, 2001), 7 in *Solanum lycopersicum* and 7 in *Asteridae* and tobacco (Liu et al., 2014; Song et al., 2019) and pepper (Jing et al., 2020). Nine and ten *Hsp90* genes were identified in rice (Hu et al., 2009) and Populus (Zhang et al., 2013), respectively. Recent studies have shown that Hsp90 has a vital role in plant’ acclimation to various biotic and abiotic stresses (Di Donato and Geisler, 2019). The *HSP* gene family were considered to be involved or induced in cold environments (Krishna et al., 1995); it has the potential to protect plant biological systems from cold damage by preventing freezing-induced protein denaturation (Yan et al., 2006). Many studies reported that the low-temperature stress activates the gene expressions of both chaperonins and Hsps, particularly Hsp90, Hsp70, and the *smHsps* (Wang et al., 2004). In *Brassica napus*, the expression of Hsp90 was upregulated under cold stress (Krishna et al., 1995; Gill et al., 2015). Likewise, rice also exhibited the up-regulated expression of *OsHsp90-2* and *OsHsp90-4* under drought, cold, and heat stress (Hu et al., 2009). Liu et al. (2022) reported that in rubber trees (*Hevea brasiliensis* Müll. Arg.) the expression of the *HbHsp90.1* gene was significantly up-regulated at 4°C for 6 h of cold stress treatment. Similarly, Song et al. (2019) observed an identical trend in the tobacco plant, where *NtHsp90-4*, *NtHsp90-5* and *NtHsp90-9* showed high levels of transcription at 4°C for 6 to 12 h duration under cold stress. Increased Hsp90 protein accumulation was observed in winter wheat under cold stress (Vítámvás et al., 2012).

*Brassica oleracea* var. *capitata* (Cabbage) is a popular vegetable crop cultivated all over the world and contains a variety of nutrients and health-promoting compounds (Lv et al., 2020). Temperature required for optimal cabbage growth is 17°C, while its normal growth and development decreased when temperature falls below 10°C; further it can withstand up to –5°C for short phase of cold exposure, prolonged exposure leads to cell death (Food and Agricultural Organization of the United Nations (FAO), 2007). This investigation executed a genome-wide analysis of the *Brassica* Genome Network database using Hsp90 protein sequences of *Arabidopsis thaliana*. We analyzed the gene structure, phylogenetic tree, chromosomal location, homologous gene pairs, conserved motifs, collinearity analysis, sectioning pressure and cis-elements of the cabbage *Hsp90* gene promoter by utilizing bioinformatics approaches. Moreover, this study explored the relevant roles of cabbage *Hsp90* gene, provides a vibrant approach for molecular breeding and develop a better understanding for its tolerance responses under cold conditions.

## Materials and Methods

### *Hsp90* Gene Family Identification in *Brassica oleracea*

To extract the *Hsp90* gene from the *B. oleracea* genome, “the *Arabidopsis Hsp90* gene was used as a query in the online database Phytozome 11.0 (Goodstein et al., 2012).^1^ The genome, CDS, protein and 1.5 kb upstream promoter region of the *Hsp90* gene were extracted and used as a query against the NCBI database^2^ to identify conserved domains and discard *Hsp90* domains missing genes. To ensure the presence of Hsp90 specific domains, all non-redundant protein sequences were checked using manual and Pfam databases, and 12 *BoHsp90* genes were finally found.

### Physical Properties and Position on Chromosomes

To predict the protein physical properties of *B. oleracea* Hsp90 (*BoHsp90*), “the ExPASy tool is utilized.^3^ The phytozome was used to obtain information about gene start-end position, the number of amino acids, and the chromosome location of the *BoHsp90* family. Plot phenograms^4^ indicate the location of each *BoHsp90* gene on its chromosome. Using standard parameters in the ExPASy program, the protein sequence of the Bo*Hsp90* gene was determined^5^“(Gasteiger et al., 2003).”

### Structural Analysis and Phylogenetic Relationship and Motif Analysis

Using the CDS and genome sequences of the related *BoHsp90* genes, construct gene structures in the Gene Structure Display Server to find exons-introns within genes (Hu et al., 2015)^6^. Multiple alignments of Hsp90 protein sequences of *B. oleracea*, *Brassica rapa, B. napus*, cucumber, tomato, potato, soybean, *Vitis vinifera*, *Zea mays, Arabidopsis,* rice, tobacco, cotton, *Medicago truncatula*, *Elaeis guineensis*, *Nelumbo nucifera*, *and P. bretschneideri* were performed by MEGA 7.0 with default parameters. Then, based on the alignment results, a phylogenetic tree was constructed using the maximum likelihood method with the following parameters: partial deletion, and 1,000 bootstrap tests. MEME is used to carry out the conserved sections of the *BoHsp90* gene (Bailey et al., 2006)^7^ and the Pfam database was used to screen their genomic assemblies (Punta et al., 2012).^8^

### Synteny Analysis of *Hsp90* Genes in *Brassica oleracea*, *Arabidopsis*, and *Brassica rapa*

By using a software MCScanX, a syntenic study of the *Hsp90* gene in *B. oleracea*, *Arabidopsis*, and *B. rapa* was performed to better understand the syntenic interactions between *A. thaliana*, *B. oleracea*, and *B. rapa* (Wang et al., 2012). It was used to check the *Hsp90* genes synteny in, *Arabidopsis*, *B. oleracea* and *B. rapa*.

### Cis-Regulatory Element Prediction for *Brassica oleracea Hsp90* Gene Promoters

The promoter sequence (1.5 kb DNA sequence in the start codon stream) of each *Hsp90* gene was derived from the *Brassica* genome. “Using the PlantCARE database, the cis-acting regulatory element then examines the promoter of each *B. oleracea Hsp90* gene^9^ (Plantcare; Lescot et al., 2002). The anticipated cis-acting regulatory elements were categorized based on their regulatory functions.”

### Gene Duplication, Ka/Ks Calculation and Positive Selection Analysis

Segmental and Tandem duplication occurred when two closely related *BoHsp90* genes were discovered on the same or different chromosomes.“Additionally, the gene duplication events *BoHsp90* genes and also synonymous substitution rate (Ka) and the non-synonymous substitution rate (Ks) were calculated using an online tool and computed their ratios.^10^ The divergence time (T) for *B. oleracea* was calculated by using T = ks/2r where R 1.5 × 10^−8^; Koch et al., 2000).” The ratio of Ka/Ks evaluates the selection pressure of *BoHsp90* repeated genes. When the ratio of Ka/Ks is>1, <1, or = 1, it is considered a positive, negative, or neutral selection. “The amino acids in the BoHsp90 protein that are under selective pressure are then clarified using the Selection 2.2 tool.^11^ The maximum likelihood test from the Bayesian inference method is used to calculate the offset ratio between codons in an aligned sequence (Yang, 1997).” Different kinds the selection type appears in the selection result, and evaluate by scale color.

### Transcriptomic Data Analysis

Gene expression patterns of cabbage in different tissues (roots, stems, flowers, leaves, scales, bulbs and callus) were retrieved from the NCBI database^12^ based on FPKM expression data with accession number GSE42891 (Yu et al., 2014). Finally, an expression heatmap was created using the TBtools software.^13^

### Plant Materials and Stress Treatments

The cabbage (cultivar Yingchuan) seeds were planted in plug trays in moistened mixed soil (peat moss) were provided by School of Horticulture, Anhui Agricultural University, Hefei, China. Seedlings were germinated and cultured for 5 to 6 weeks in a glass chamber at 22°C on a 16/8 h. (light/dark) cycle. Then, they are treated at low temperatures (−2°C, 0°C, and 4°C) for 6, 12 and 24 h (Ahmed et al., 2015). Plants that had not been treated were cultivated normally. At 6, 12, and 24 h following treatment, all true leaves were sampled and quickly put into liquid nitrogen and stored at −80°C for RNA extraction and synthesis of cDNA.

### RNA Isolation, cDNA Synthesis and qRT-PCR Analysis to Validate the *BoHsp90* Genes Expression

The Novaprotein reagent or RNA isolation kit for RNA extraction from cabbage leaves and quantification using a NanoDrop spectrophotometer (Thermo Scientific). “The superscript III Reverse Transcriptase (Novaprotein) according to the manufacturer’s guidelines, and the cDNA was dilute to 100 ng/l with ddH2O for further analysis. Quantitative RT-PCR was performed with the CFX96 Real Time System (Bio-Rad) with 96 well plates were used, and each well contained a total reaction mixture (20 μl) consisting of 10 μl of Power SYBR Master mix, 1 μl primer mix each F/R primers +6 μl ddH2O) and 2 μl cDNA.” The transcript level was normalized with a housekeeping gene *BolActin*. The 3 biological replicates were used for each *BoHsp90s*. The primers sequence for each gene were designed by using Primer5 software as shown in Supplementary Table S2.

## Results

### Identification of the *Hsp90* Gene Family in *Brassica oleracea*

Using the *A. thaliana* Hsp90 protein sequence (BAB09283.1) as a query sequence, by using the online database phytozome^14^ to find *Hsp90* genes in the *Brassica* genome. “The blast outcomes of *A. thaliana* in *Brassica* gave 18 hits in phytozome.” The signature *BoHsp90* domain for each protein was confirmed using SMART Pfam^15^ (Punta et al., 2012). Additionally, NCBI web-based conserved domain search tool^16^ (Marchler-Bauer et al., 2015) was used to verify the conserved domain, and the protein sequences lacking the *BoHsp90* domain were removed. During an extensive search of the *Hsp90* gene, 12 putative genes were found. The annotation of known *Brassica BoHsp90* family members were carried out, based on their chromosomal wise arrangements (*BoHsp901-12*). The protein sequences of *BoHsp90* genes extended from 44 (*BoHsp90-12*) to 1819 (*BoHsp90-11*) amino (aa) residue with molecular weight (MW) range of 46.93–201.60 kDa. All 12 Hsp90 members have an average length of 773.4 aa and an average MW of 87.92 KDa furthermore, 41.66% (5 out of 12 genes) of *BoHsp90* genes had high pI values (more than 5), while 58.33% (7 out of 12 genes) had pI values below 5. The predicted subcellular localization includes nuclear, endoplasmic reticulum and cytoplasmic. These results revealed that the maximum number of genes are found in the cytoplasm and percentage found in the nuclear (Supplementary Table S1).

### Phylogenetic Relationship of the *BoHsp90* Genes in *Brassica oleracea*

**Figure 1:**
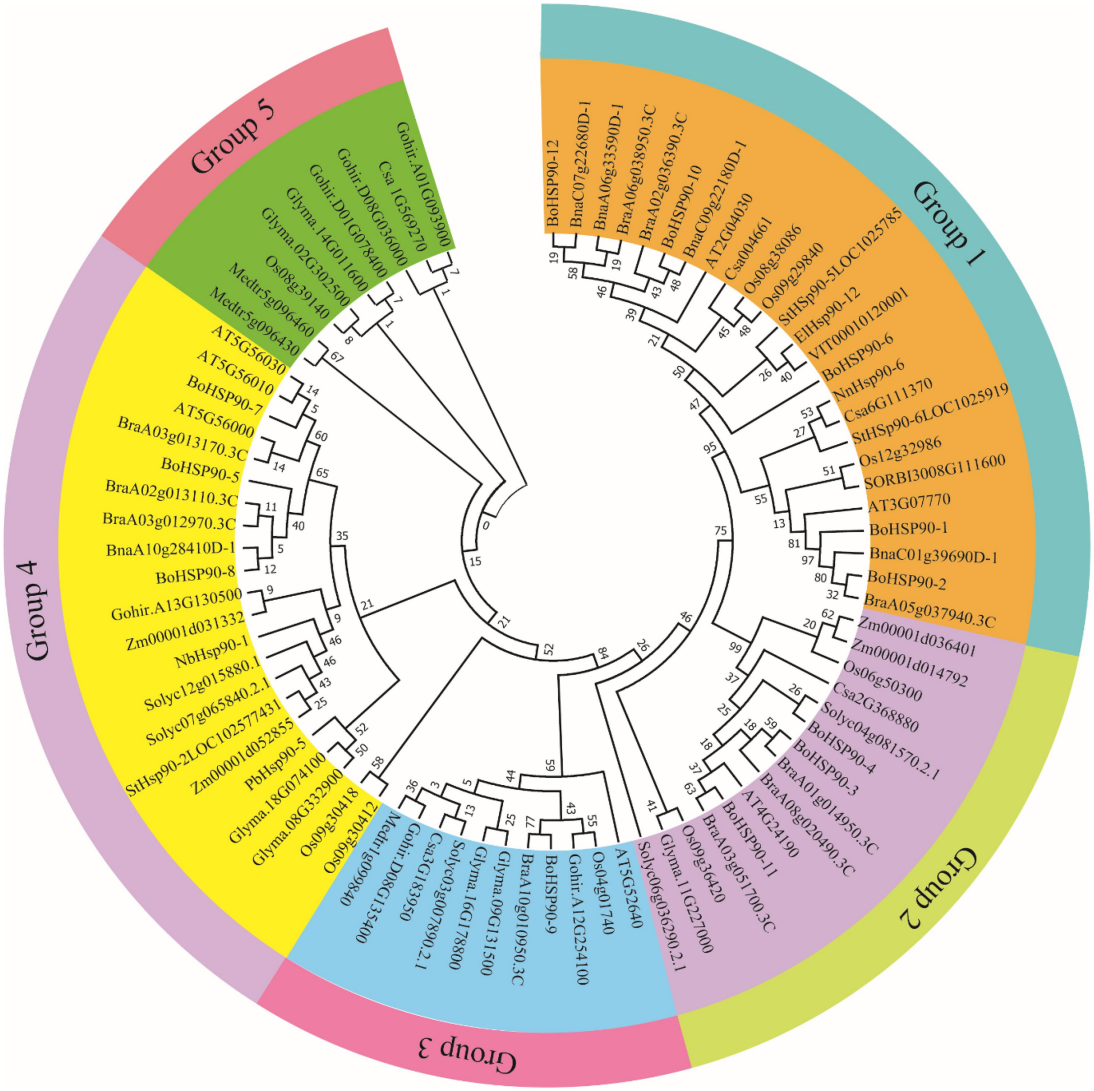
Phylogenetic tree of the Hsp90 proteins from *Brassica oleracea*, *Brassica rapa*, *Brassica napus*, tomato, cucumber, potato, *Vitis vinifera*, *Sorghium bicolor*, cotton, tobacco, *Arabidopsis*, rice, *Zea mays*, soybean, *Medicago truncatula, Elaeis guineensis*, Chinese white pear (*Pyrus bretschneideri*) and *Nelumbo nucifera*”

To study their evolutionary relationship, the predicted protein sequence of BoHsp90 was employed to create a rootless phylogenetic tree. The phylogenetic study of the *Hsp90* genes distributed into five groups (G1, G2, G3, G4, and G5) containing *B. oleracea*, *B. rapa*, *B. napus*, tomato, cucumber, potato, *V. vinifera*, *Sorghum bicolor*, cotton, tobacco, *Arabidopsis*, rice, *Z. mays*, soybean, *M. truncatula, E. guineensis*, Chinese white pear (*Pyrus bretschneideri*) and *N. nucifera*. Among them G1 is the largest group with a total of 25 members, having *B. oleracea* (4 genes), B. *napa* (4 genes), *B. rapa* (3 genes), rice (3 genes), cucumbers (2 genes), potatoes (2 genes), *A. thaliana* (2 genes), 1 *V. vinifera* (1 gene), *E. guineensis* (1 gene), *N. nucifera* (1 gene) and one from sorghum. The second largest group, G4, comprises of 22 members, having *B. oleracea* (3 genes), *B. rapa* (3 genes), *B. napus* (1 gene), *A. thaliana* (3 genes), *soybeans* (2 genes), tomato (2 genes), rice (2 genes), *Z. mays* (2 genes), potato (1 gene), tobacco (1 gene), cotton (1 gene), and one *P. bretschneideri*. The G2 group contain 15 members, among which 3 in *B. oleracea*, 3 in *B. rapa*, one in *A. thaliana*, 2 in *Z. mays*, 2 in rice, 2 in tomato, one in cucumber, and one in soybean. While G3 comprised of 10 members as 2 in cotton, 2 in soybean, one in *B. oleracea*, *B. rapa*, *A. thaliana*, cucumber, tomato, and *M. truncatula*. G5 composed of 9 members and considered as a small group, having 3 in cotton, 2 in soybean, 2 in *M. truncatula*, one in cucumber and rice member (Figure 1).

### Structural Analysis of the *BoHsp90* Genes

**Figure 2:**
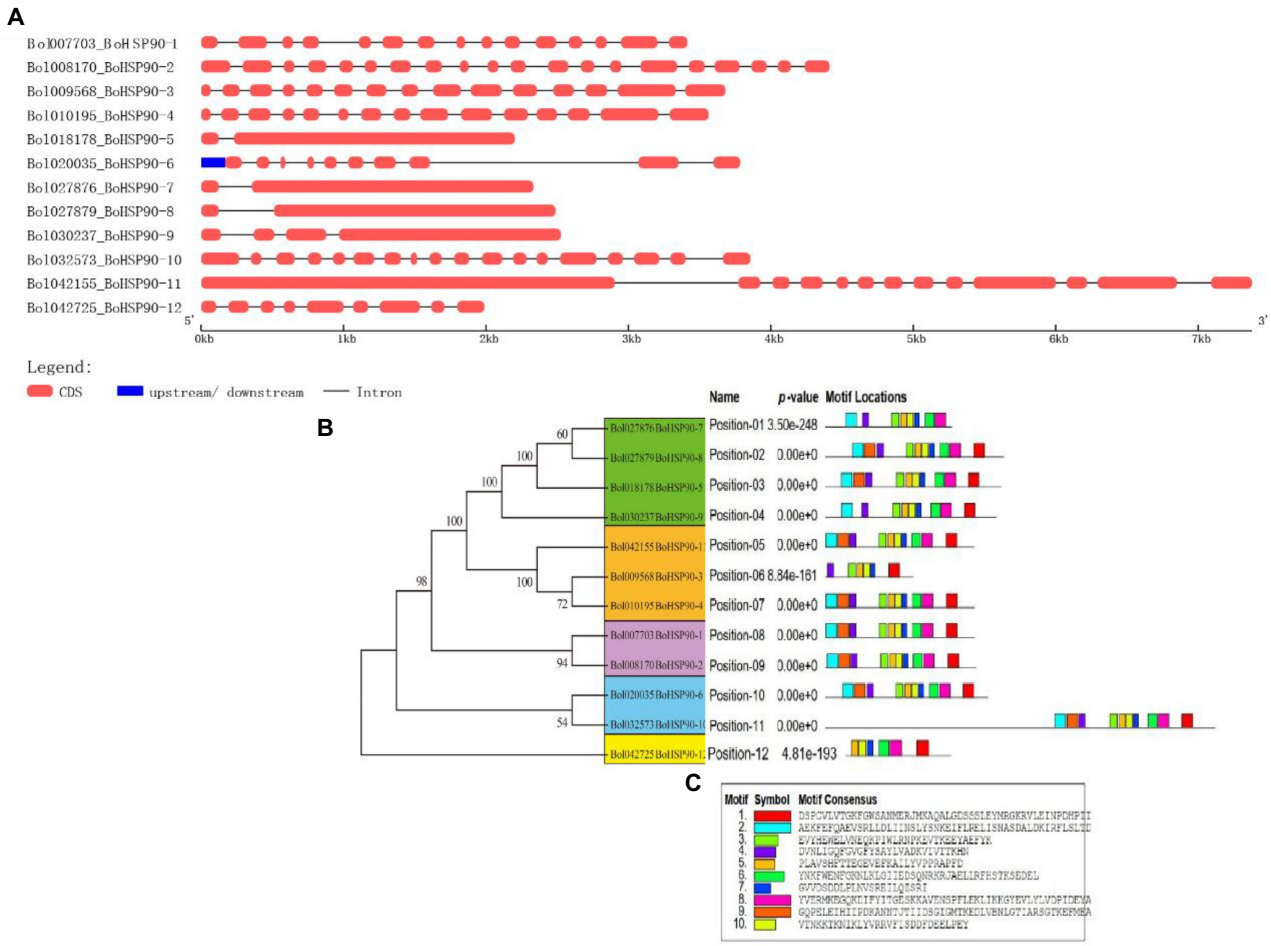
Gene structure and conserved motif analysis of *BoHsp90* genes. **(A)** Gene structure of the *BoHsp90* genes in cabbage. **(B)** Motif composition of *BoHsp90*. **(C)** A conserved motif in *BoHsp90* gene was detected with MEME. Ten different motifs are represented by variously colored boxes.

The arrangement of the *BoHsp90* genes was plotted using the online tool GSDS based on their coding sequence. Within each *BoHsp90* gene, “(Figure 2) displays the comparative lengths of introns and the conservation of corresponding exon sequences.” All these genes have between 1 and 19 introns. However, half of the *BoHsp90* genes contain 1 to 14 introns, while another half of *BoHsp90* genes contain different numbers of introns likewise *BoHsp90-2* has 19 introns, *BoHsp90–10* contains 17 introns, 12 in *BoHsp90-11*, 9 in *BoHsp90-6*, *BoHsp90*-12 has 8 introns and 3 introns in the *BoHsp90–9.* The position and length of introns also vary widely. In order to better understand the structural properties of BoHsp90 protein, 10 conserved motifs in the protein were identified using the online program MEME motif search tool and the distribution of these conserved motifs in BoHsp90 protein was explored.” The findings revealed that the majority of the genes comprised 10 conserved motifs. There are similar motifs in closely related genes, indicating similar functions in the *BoHsp90* gene family. The *BoHsp90-3* and *BoHsp90*-12 contain six motifs, *BoHsp90-7* has eight and 9 motifs were found in *BoHsp90-9*. According to these findings, structural motif alignment varies between members of the *BoHsp90* family genes but is similar within closely related genes (Supplementary Table S5).

### Chromosomal Distribution, Collinearity Analysis of the *Hsp90* Gene Family in ***Brassica oleracea***

**Figure 3:**
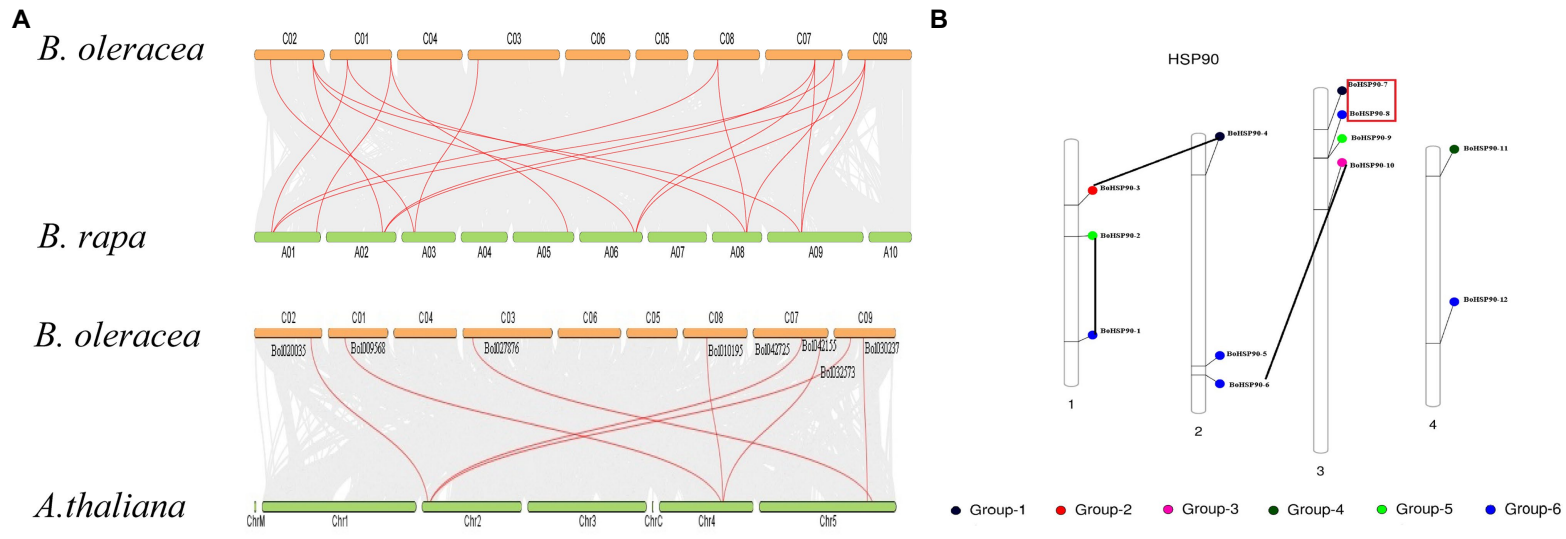
Chromosomal location and duplication of Cabbage (*B. oleracea) Hsp90* genes. **(A)** Red boxes indicate tandem duplications, and black lines indicate segmental duplications. **(B)** Collinearity relationship or Synteny analysis of *Hsp90* genes in *B. oleracea*, *B. rapa*, and *Arabidopsis*.

The chromosomal location map shown in (Figure 3) was created to specify the chromosomal orientation and deletion site of the *BoHsp90* gene. The findings revealed that a total of 12 genes were linked to four chromosomes in cabbage. Of these, 83% of the *BoHsp90* gene (10 loci) are present on three chromosomes in cabbage. Chromosomes 3, 2, and the more repressed *Hsp90* members each have three and four genes, while chromosome 4 contains two genes. Furthermore, we also explored the collinearity relationships between the Cabbage *BoHsp90* genes and related genes from 2 representative species including *A. thaliana* and *B. rapa*. A total of 12 cabbage *Hsp90* genes have collinearity relationships with 5 *A. thaliana* genes. 10 *B. rapa* genes.

### Duplication, Ka/Ks and Evolutionary Analysis of the *BoHsp90* Genes in ***Brassica oleracea***

The Ka/Ks ratio can be used to practice the selection process history of coding sequences “(Li et al., 1981). To explore the bias of duplicated *BoHsp90* members, the Ka, Ks and Ka/Ks ratios of Hsp90 paralogous members were determined.” The Ks values were used to estimate each gene’s fraction. “All of the paralogous Hsp90 pairs had a Ks value ranging from 0.1 to 0.3, which was made up of cabbage duplication events. In addition, the third pair of *BoHspP90* genes obtained duplications from 0.28 to 13.05 Mya (Table 1). The Ka/Ks ratio can also be used to determine selection pressure. The value of Ka/Ks1, greater than or equal to one, shows that the paralogous pair was subjected to, positive, purifying and neutral selection, or rare selection, respectively (Juretic et al., 2005).” Consequently, all the values were smaller than one, the representative that the *BoHsp90* genes changed largely under the influence of purifying selection (Table 1). “Consistent evolution of amino acid positions is imperative for conservation of function and protein structure.” Certain amino acid identifications may be useful in elucidating selection pressures and assessing the relevance of specific amino acid interactions in Hsp90 protein interactions. The MEC model’s positive selection test for Hsp90 protein estimates the selection pressure of a given coding region. Our research shows that 38.76% (271 out of 699) amino acids are purifying selected, while the rest are affected by the neutral process (Figure 4). The results clarify the evolution all gene pairs are affected by purification selection.

**Table 1:** Duplicated *BoHsp90* genes in cabbage.

#	Paralogous pair	Ka	Ks	KaKs	Time (Mya)
1	*Bol027876(BoHsp90-7)-Bol027879(BoHsp90-8)*	0.007	0.186	0.042	6.21
2	*Bol009568(BoHsp90-3)-Bol010195(BoHsp90-4)*	0.023	0.309	0.076	10.32
3	*Bol020035(BoHsp90-6)-Bol032573(BoHsp90-10)*	0.110	0.391	0.281	13.05

**Figure 4:**
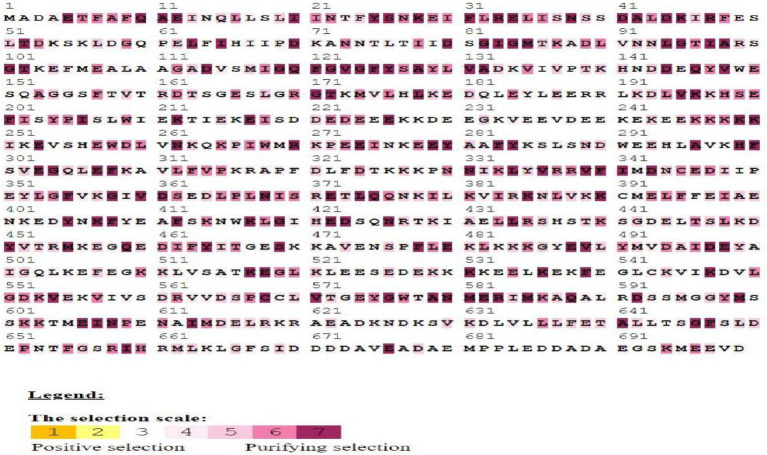
Selection pressures among *Hsp90* gene sequences using mechanistic-empirical combination (MEC) model of selection online tool. Gray and white highlights represent neutral selection, and purple highlight represents purifying or negative selection on codons.”

### Analysis of the Promoter Region of *Brassica oleracea Hsp90* genes

**Figure 5:**
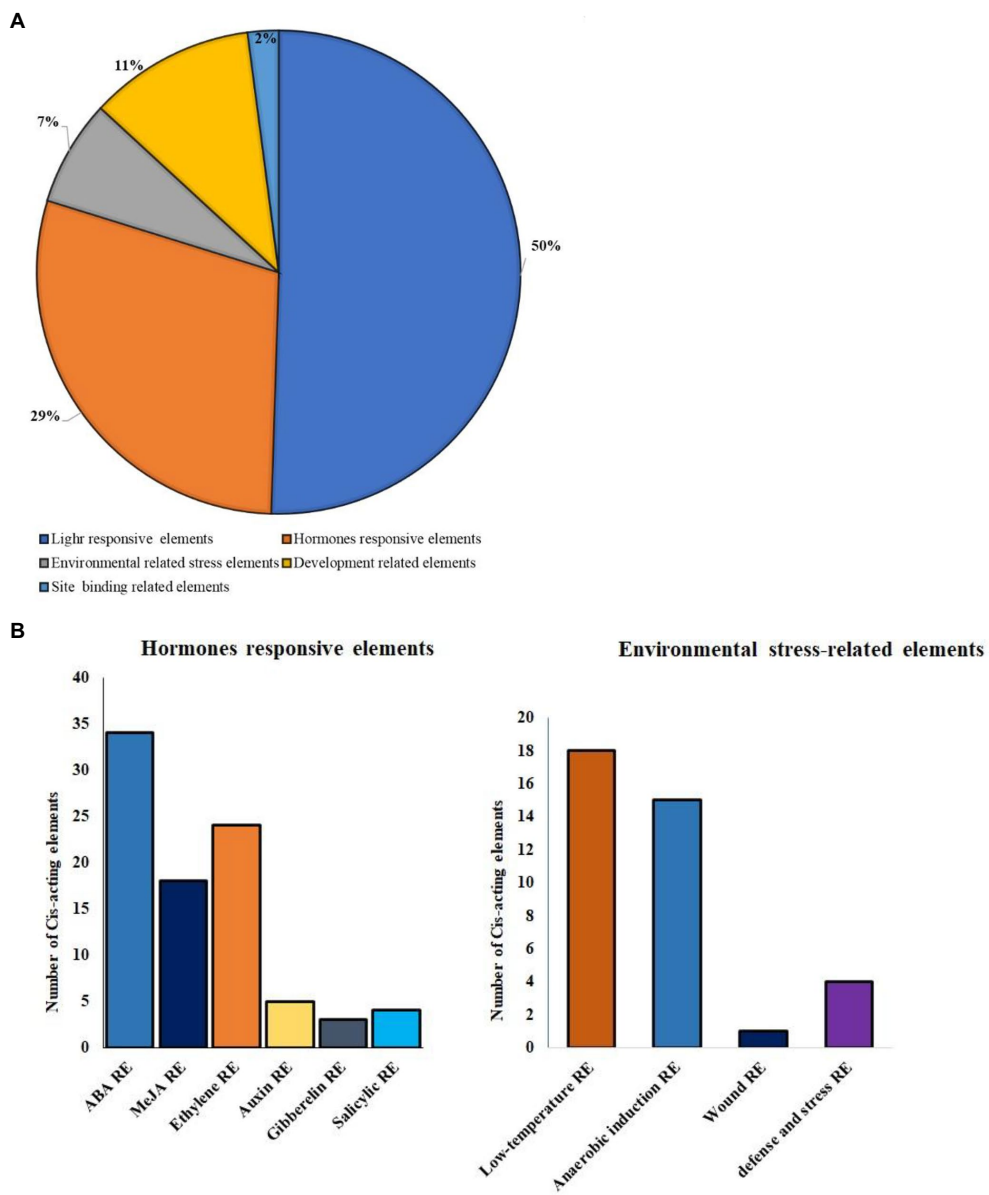
Distribution of cis-elements in the promoter of *B. oleracea Hsp90* genes. **(A)** The proportion of cis-elements in the promoters of *B. oleracea Hsp90* genes are are shown in a pie chart. **(B)** Different hormones responsive elements and environmental stress-related elements in *12 BoHsp90* genes cis-elements regions.

Each *BoHsp90* gene was isolated from the *Brassica* plant genome, and the cis-regulatory region (1,500 bp upstream) was assessed using PlantCARE to better understand its possible functions and regulatory processes. The 12 *BoHsp90* promoter region of contain 50% of light-responsive elements, responsive to hormones 29%, environmental responsive elements 7%, development-related elements contain 11% and site binding elements 2% (Figure 5A). “There are various transcription factor binding sites that govern hormones such as gibberellic acid (GA), abscisic acid (ABA), jasmonic acid, auxin, silicic acid, and ethylene. In addition to the previously mentioned regions (Figure 5B) or by stress response (defense, heat, low-temperature stress, etc). The *BoHsp90-3* promoter region contains a maximum of 11 ABRE-related elements and 3 for jasmonic acid-responsive elements (Supplementary Table S3).

### Tissue-Specific Expression Profile Analysis of *Brassica oleracea* the *Hsp90* Genes

**Figure 6:**
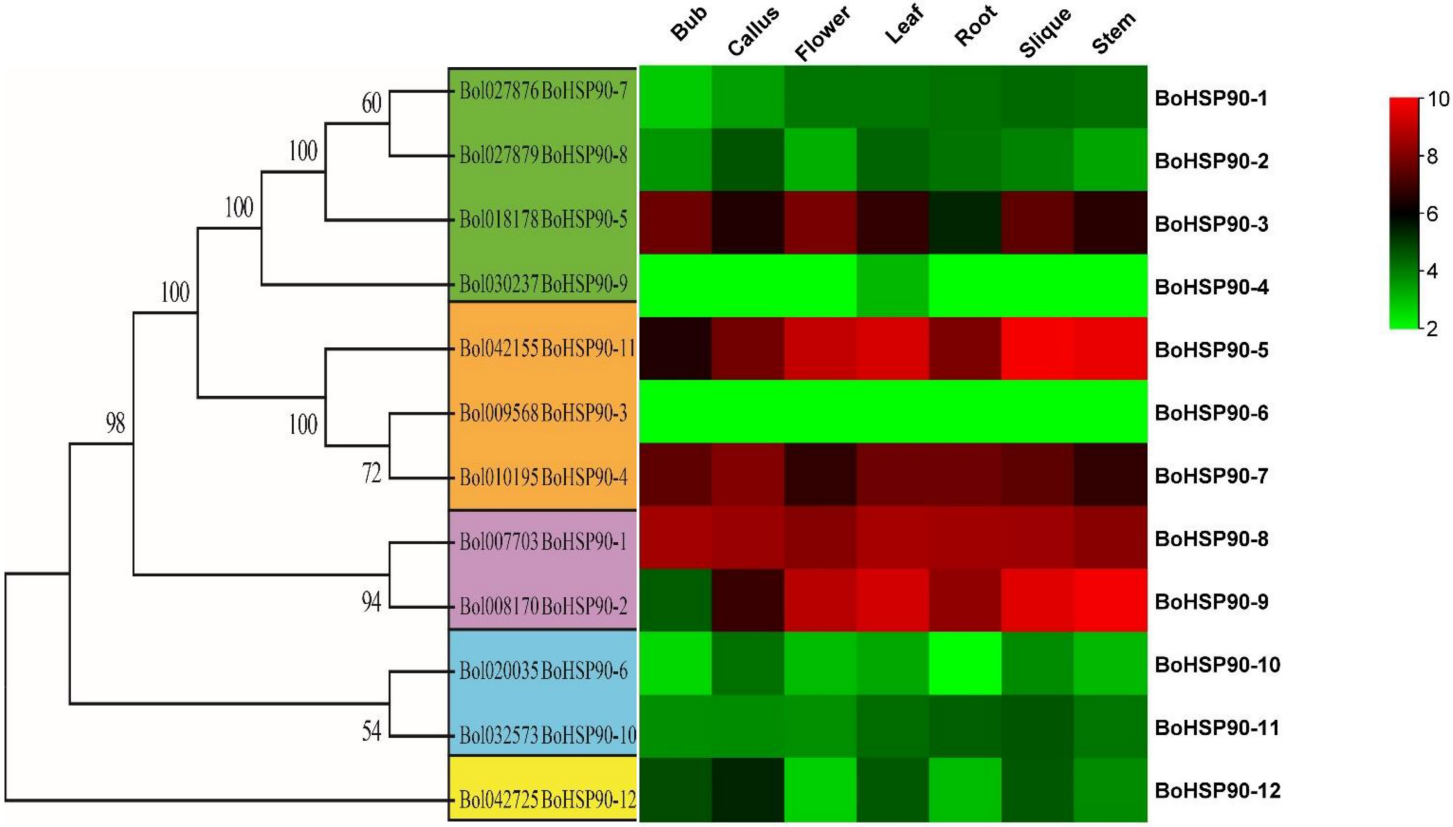
The heat map of 12 *BoHsp90* genes was constructed using Tbtools software. The FPKM values were log2 transformed and mean-centered by genes using the scale 10 for minimum expression (red color) and 2 (green color) for maximum to make a heat map visible for each cabbage tissue.

The expression profile of the *BoHsp90* gene was generated using RNA-seq data from seven tissues (root, stem, leaf, shoot, flower, callus, and silique). The expression pattern was derived from publicly accessible Illumina RNA-Seq data for Cabbage at NCBI (Liu et al., 2014).^17^ The expression of the *BoHsp90* gene was found to be higher in stem and silique tissues than in other tissues (Figure 6). The 12 predicted *BoHsp90* genes were found to be active in at least one of the six tissues. The expression of *BoHsp90–9* and *BoHsp90–5* was found to be high in the stem, silique, leaves, and flowers. Two genes were upregulated in stem, silique, leaves, and flowers (*BoHsp90-9* and *BoHsp90-5*). “Target genes involved in plant growth and development have been shown to be affected by genes that are highly expressed in plant tissues or organs. In this study, we found development-related *BoHsp90* genes in the cabbage genome at the transcription level, and two genes (*BoHsp90–5*, and *9*) were selected in stem, leaves, silique, and flowers that were upregulated, downregulated, or not different in other tissues according to normalized FPKM values. (Supplementary Table S4).

### Expression Profiles Analysis of *Brassica oleracea Hsp90* Genes Under Cold Stress

**Figure 7:**
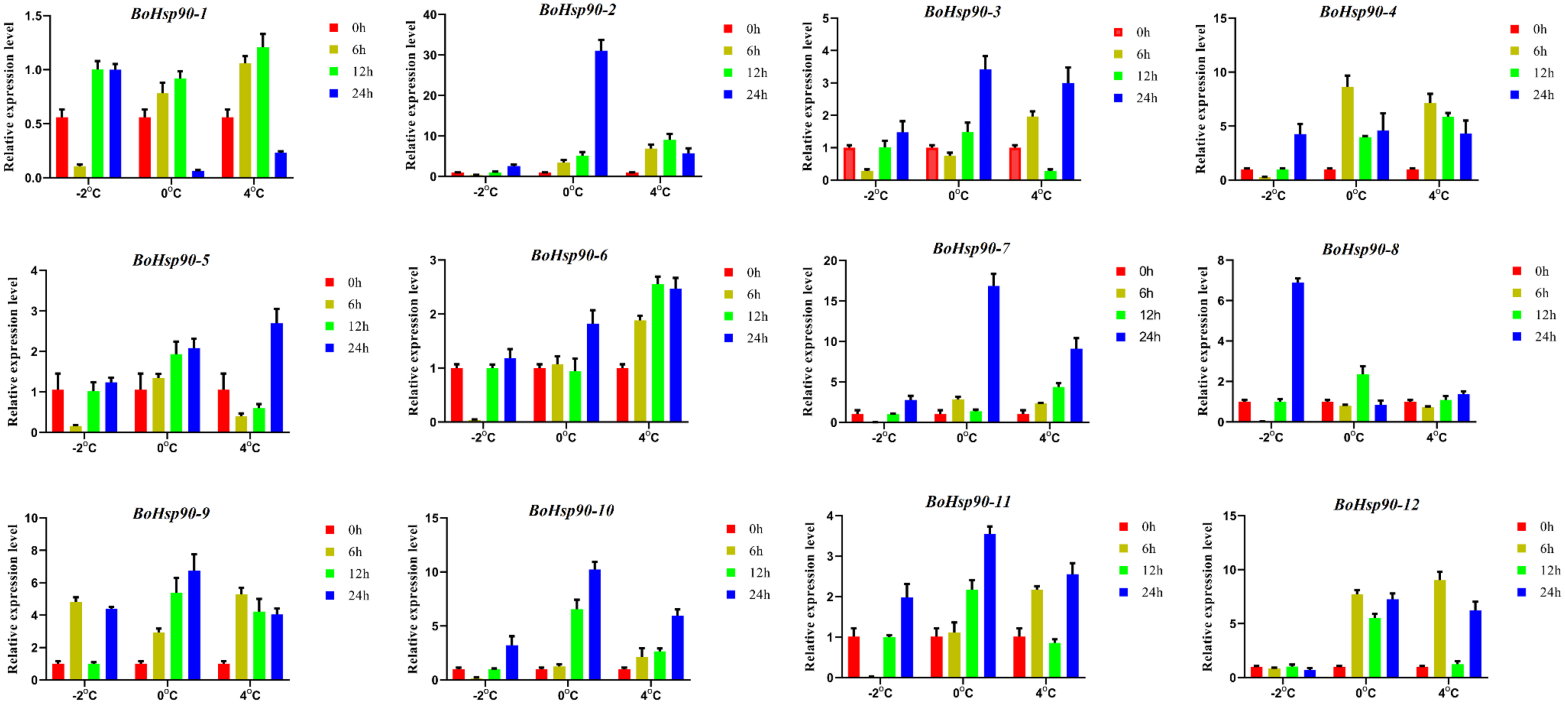
Relative expression levels of 12 *Hsp90* genes in *B. oleracea* leaves by qRT-PCR after 0 h (control), 6 h, 12 h and 24 h after treatment of cold stress at −2°C, 0°C, and 4°C. The expression levels of *Hsp90* genes were normalized to *BoActin* using as an internal control. Error bars indicate the standard error (SE) between three replicates.

To confirm the expression patterns determined by the qRT-PCR was employed to analyze the expression patterns of 12 different *BoHsp90* genes under cold stress conditions. Different expression profiles of 12 *BoHsp90* genes in response to cold stress were identified. (Figure 7). The result showed that most of the genes showed high expression levels at 4°C for 12 h intervals. Besides the expression level of *BoHsp90-2, BoHsp90-3, BoHsp90-7, BoHsp90-9, BoHsp90-2, and BoHsp90-11* were highly upregulated at 4°C after 12 h exposure of cold stress. we have further noticed that expression patterns of genes, including, *BoHsp90-2*, *BoHsp90-3, BoHsp90-4*, *BoHsp90-5*, *BoHsp90-7*, *BoHsp90-8*, *BoHSP90-10*, and *BoHSP90-12* were gradually downregulated as time passed. Similarly, *BoHSP90-1*, *BoHsp90-4, BoHsp90-6, BoHsp90-9* were highly upregulated at −2 and 0°C for 24 h intervals, but these genes showed downregulation trend at 6 h. The findings from this study exhibited that the *B.oleracea Hsp90* genes family, which are responded to cold stress promptly and with a long response time.

## Discussion

The unpredictability of global temperatures is followed by an increase in the frequency of cold spells, which is harmful for crop growth and effected the yields, and understanding the response of plants to low-temperature stress is very important for plant growth (Ding et al., 2019). When the temperature fluctuates (rises and falls) in comparison to normal, the physiological and biochemical reactions in the organism are inhibited, and cold stress inhibits the majority of protein and mRNA transcription. However, temperature stress (high and low) typically alters gene expression in plants, and an important type of highly conserved protein known as HSPs is rapidly synthesized. The Hsp90 is a chaperone protein that is highly conserved in prokaryotes and all eukaryotes and is involved in the cell cycle, signaling, and other key biological processes (Pearl and Prodromou, 2006). HSPs were first identified in the salivary gland chromosomes of Drosophila larvae (Ritossa, 1962). The function of *Hsp90* genes has been extensively studied in animal and fungal systems to date, but not widely studied in plants. However, with the continuous advancement and wider application of genome sequencing technologies, more and more plant genome data for Hsp90 have been released recently (Kersey, 2019). The *Hsp90* gene was found to be involved not only in stress signal transduction, receptor folding, transcription factors and kinases, and physiological processes in plants (Chory and Wu, 2001; Mach, 2009; Cha et al., 2013; Han et al., 2015) but also in assisting cell survival in stressful situations (Jackson et al., 2004). The *Hsp90* gene family has been identified in two model plants, *Arabidopsis* and rice (Hu et al., 2009). Subsequently, the *Hsp90* gene family was discovered in some major vegetable crops, such as tomato (Liu et al., 2014) and pepper (Jing et al., 2020). Cabbage is a very important vegetable crop cultivated in many areas worldwide. *B. oleracea* seedlings must be vernalized at 0–10°C and then licked, blossomed, and bore fruit under long sunshine and moderate temperature. Due to its characteristics of different maturity, suitable for different seasons, wide adaptability, and strong stress resistance, *B. oleracea* occupies an important position in the annual production of vegetables (Zhu et al., 2017). But the low temperature was one of the most important limiting factors for the wintering cultivation of *B. oleracea* in open fields; during winter *B. oleracea* often suffers from low temperatures and even sub-zero temperatures. The low-temperature conditions make its yield and quality decline. However, *Hsp90* gene family has not been characterized in cabbage (*B. oleracea*). Therefore, we investigated the whole genome of the *BoHsp90* gene family in cabbage, including phylogeny, chromosomal location, gene structure, conserved motifs, and expression profiles. A total of 12 *Hsp90* genes in cabbage were identified in this study, which are greater than other plant species such as 7 in *Arabidopsis* (Krishna and Gloor, 2001), 9 in rice (Hu et al., 2009), 10 in poplar (Zhang et al., 2013), 7 in tomato (Liu et al., 2014), and 7 in pepper (Jing et al., 2020). This could be the one of the main reasons for the difference in cold tolerance ability among different plant species. In this study, using the *Arabidopsis* Hsp90 protein sequence, 12 *Hsp90* genes were retrieved from the *Brassica* plant genome. These Hsp90 family proteins are vital in acclimatizing unfavorable environmental conditions through regulation and maintenance of physiological mechanism of cabbage plant (Zhang et al., 2021). The various physicochemical properties of the Hsp90 family proteins imply that there is a great deal of diversity among members, which will help in further research into the functions of *Hsp90* genes. Different features of the Hsp90 protein family, such as molecular weight, isoelectric point, and the number of exon-introns, were investigated in cabbage in this work. “Furthermore, all Hsp90 proteins in *B. oleracea* were acidic nature, which was similar with findings from *A. thaliana*, tomato, and other plants (Liu et al., 2014). These results suggested that Hsp90 proteins were conserved across plant species. The amino acid sequences of Hsp90 family proteins provided phylogenetic information based on their basic structures. In different *BoHsp90* gene sequences, we found different numbers of introns (1–19 introns). The fewer introns a plant has, the better its capability to respond to various environmental stimuli and developmental processes (Jin et al., 2014). Long-term evolution has resulted in a high number of introns in *BoHsp90s*. Song et al. (2019) revealed that conserved motif analysis of *Hsp90* genes in tobacco*, Nitab4.5_0001622g0050* and *Nitab4.5_0003328g0120* consist of fewer motifs, hinting that it had lost sequencing parts during evolution. Referring to previous investigations, the cabbage *Hsp90* genes’ conserved motif analysis, *Bol009568.BoHsp90-3* and *Bol42725.BoHsp90-12* shown fewer motifs, which might be due to misplaced sequence sections during evolution. In addition, *BoHsp90-3* and *BoHsp90-12* contain 6 motifs, *BoHsp90-7* has 8 motifs, and 9 motifs were found in *BoHsp90-9*. These findings suggested that the structural motifs are aligned differently in various members of the *BoHsp90* family genes but are similar within closely related genes.

Gene duplication is a crucial mechanism in the evolution of gene families (Yang et al., 2008). In cabbage, at least three pairs of duplicated genes were discovered, representing that gene replication may have occurred throughout the evolution of the *Hsp90* genes. Phylogenetic analysis is frequently used to learn more about species’ evolutionary changes and to discover the orthologs and paralogs within species (Moreira and Philippe, 2000). An unrooted phylogenetic tree was constructed using protein sequences from *B. oleracea*, *B. rapa*, *B. napus*, tomato, cucumber, potato, *V. vinifera, Sorghium bicolor,* cotton, tobacco*, Arabidopsis*, rice, *Z. mays*, soya bean, *E. guineensis*, Chinese white pear (*Pyrus bretschneideri*) and *N. nucifera*. Hsp90*s* can be split into five groups based on evolutionary study. Protein function is determined by its structure (Skolnick and Fetrow, 2000). According to the collinearity relationships between the *B. orleacea BoHsp90* family genes and 2 representative plant species including A. thalian and *B. rapa*, only three pair of paralogous pair (*Bol027876BoHsp90-7-Bol027879.BoHsp90-8, Bol01019568.BoHsp90-3-Bol010195Hsp90.4 and Bol020035.BoHsp90-6-Bol032573.BolHsp90-10*) were perceived and segmental duplication were not found, representing that enlargement of *Hsp90* gene in *B. oleracea* was mainly caused by tandem duplication genes. Only four *B. oleracea Hsp90* genes *Bol7703-BoHsp90-1, Bol008170.BolHsp90-2, Bol018178.BoHsp90-5 and Bol027879.BoHsp90-8* was conservative and not collinearity relation with *Hsp90* genes in *B. rapa* and *Arabidopsis*. However, eight *B. oleracea Hsp90* genes have collinearity relationships with 5 genes of *A. thaliana* and 10 genes of *B. rapa*. These findings suggested that the *Hsp90* gene family expands differently in each species, as this pattern was also noticed in other plant gene families (Huang et al., 2016). Furthermore, promoter analysis revealed that the promoter of the *B. oleracea Hsp90* gene contained various regulatory elements related to light-responsive elements, responsive to hormones, environmental responsive elements, development-related elements, and site binding elements, among others, implying that these regulatory elements actively engage in a variety of biotic and abiotic stress responses in plants and also indicating that *Hsp90* genes are important in cell-cycle control, signal transduction, genomic silencing, protein degradation, and protein trafficking.

Recent studies have shown that Hsp90 has a vital role in plant acclimation to various biotic and abiotic stresses (Di Donato and Geisler, 2019). The *Hsp* gene family was considered to be involved or induced under cold environments (Krishna et al., 1995); it has the potential to protect plant biological systems from cold damage by preventing freezing-induced protein denaturation (Yan et al., 2006). In this study, the publicly online tissue specific expression analysis showed that the *BoHsp90* gene was found to be higher in stem and silique tissues than other tissues. Similarly, the expression of *BoHsp90–9* and *BoHsp90–5* was found to be high in the stem, silique, leaves, and flowers. While upregulated in stem, silique, leaves and flowers. These findings indicated that the functions of *B. oleracea Hsp90* genes varied across tissues. Many studies indicated that *Hsp90* gene family induced under low-temperature stress (Krishna et al., 1995; Di Donato and Geisler, 2019).

Gene expression patterns were highly correlated with their functions. The tissue-specific expression patterns at a given developmental stage are crucial for determining the gene functions in which they are involved. The expression pattern of different genes was studied in our work under diverse stresses and distinct cabbage tissues to better understand their possible significance during cold stress and development responses. The majority of the genes investigated exhibited expression in developmental tissues, indicating their function in these tissues (Figure 7). However, the *Hsp90* gene family in cabbage has not been investigated to date. Thus, to understand the functions of *Hsp90* genes under cold stress, we accomplish qRT-PCR of 12 different genes under cold stress. These results indicated that expressions of *Hsp90* genes family, *BoHsp90-1/6/8/9/11*, *BoHsp90-1*, and *BoHsp90-9* were highly upregulated at 4°C, 0°C, and -2°C respectively, while remaining were down-regulated at respective low temperatures for 6 h duration. In the same manner, gene expressions of *BoHsp90-2/3/5/6/7/9/10/11/12*, *BoHsp90-1/4/9/10/11/12*, and *BoHsp90-1* were significantly upregulated at 4°C, 0°C, and -2°C, respectively; while *BoHsp90-1/4/8*, *BoHsp90-2/3/6/7/8*, *BoHsp90-2/3/4/5/6/7/8/9/10/11/12* were downregulated at 4°C, 0°C, and -2°C, respectively, for 12 h duration. Lastly for 24 h duration, gene expressions of *BoHsp90-3/5/6/7/10/11/12*, *BoHsp90-1/4/6/9*, and *BoHsp90-1/3/4/6/9/11/12* were upregulated at 4°C, 0°C, and -2°C, respectively; while *BoHsp90-1/2/4/8/9*, *BoHsp90-2/3/5/7/8/10/11/12*, and *BoHsp90-2/5/7/8/10* were downregulated at 4°C, 0°C, and -2°C, respectively. Previous studies also reported that Hsp90 protein accumulation was found to be altered in cold-treated winter wheat followed cold treatment (Vítámvás et al., 2012). Overall, the cabbage *Hsp90* gene family was found to be differentially expressed under cold stress, suggesting that these genes play an important role in cabbage growth and development under cold conditions. We found the evolution of *Hsp90* genes in cabbage using comparative genomics analysis, and we screened putative regulatory genes associated with cold stress. The biological activities of these genes will be examined in the subsequent study, which will give an essential theoretical foundation for increasing cabbage quality.

## Conclusion

In this study, *Hsp90* family genes were identified and characterized in the *Brassica oleracea*. A total of 12 *Hsp90* genes of *Brassica* were identified and characterized using phylogenetic tree, gene structure, physicochemical characteristics, conserved motif analysis, chromosomal location, synteny, selection pressure, homologous gene pairs, and cis-elements in the promoters, which revealed a rich evolutionary history for this *Brassica* family. The expression pattern analysis of *B. oleracea BoHsp90* exhibited that *BoHsp90-2, BoHsp90-3, BoHsp90-7, BoHsp90-9, BoHsp90-10,* and *BoHsp90-11* were induced under cold stress, which indicates these *Hsp90* genes perform a vital role in cold acclimation and supports in the continual of normal growth and development process. Our findings provide a scientific foundation for more comprehensive understanding of the cabbage *Hsp90* gene family, and it will also contribute to the development of new high yielding and stress tolerant cultivars of *B. oleracea*.

## Data Availability Statement

Publicly available datasets were analyzed in this study. This data can be found here: https://www.ncbi.nlm.nih.gov/gds/?term=GSE42891.

## Author Contributions

SS and SJ conceived and designed the work. MA, QD, YL, MH, and CT performed the experiments and conduct the bioinformatics analysis. SS and JS wrote and revised the manuscript. All authors contributed to the article and approved the submitted version.

## Funding

This study was financially supported by the National Natural Science Foundation of China (31872108, 31272170) and the Key Research and Development Projects in Anhui Province (No. 202104b11020006).

## Conflict of Interest

The authors declare that the research was conducted in the absence of any commercial or financial relationships that could be construed as a potential conflict of interest.

## Publisher’s Note

All claims expressed in this article are solely those of the authors and do not necessarily represent those of their affiliated organizations, or those of the publisher, the editors and the reviewers. Any product that may be evaluated in this article, or claim that may be made by its manufacturer, is not guaranteed or endorsed by the publisher.

## Footnotes

^1^ https://phytozome-next.jgi.doe.gov/

^2^ www.ncbi.nlm.nih.gov/Structure/

^3^ http://web.expasy.org/protparam

^4^ http://visualization.ritchielab.org/phenograms/plot

^5^ http://www.expasy.org/tools

^6^ http://gsds.cbi.pku.edu.cn/

^7^ https://www.meme-suite.org/meme/

^8^ https://pfam.sanger.ac.uk/

^9^ http://bioinformatics.psb.ugent.be/

^10^ https://services.cbu.uib.no/tools/kaks

^11^ http://selecton.tau.ac.il

^12^ https://www.ncbi.nlm.nih.gov/

^13^ http://www.tbtools.com/

^14^ http://phytozome.jgi.doe.gov/pz/portal.html

^15^ http://pfam.xfam.org/

^16^ https://www.ncbi.nlm.nih.gov/Structure/cdd/wrpsb.cgi

^17^ www.ncbi.nlm.nih.gov/gds/?term=GSE42891
