# Supplementary material for: Genome-Wide Study of Hsp90 Gene Family in Cabbage (Brassica oleracea var. capitata L.) and Their Imperative Roles in Response to Cold Stress
Source: Front Plant Sci. 2022 Jun 22;13:908511. doi: 10.3389/fpls.2022.908511 (PMC9258498; doi:10.3389/fpls.2022.908511)
Supplement: Supplementary file 2 [file Data_Sheet_2.docx]

***Supplementary Materials***

| **Supplementary Table 1:** List of BoHSP90 genes primers used in the qRT-PCR analysis | | |
| --- | --- | --- |
|  | |  |
| **Gene Name** | **Forward Primer (5'-3')** | **Reverse Primer (5'-3')** |
| BoHSP90-1 | GTTGAAAGATACTGGGATTGGGAG | AGGGTCCGTAACGCTCAAATAAC |
| BoHSP90-2 | GCAAGAAAAGGGATACACAAAAGAG | TTAGTCAGTTCCCAGTCCCAGTATC |
| BoHSP90-3 | ATACCTGATGGACTACGAGGACAAG | CTTTCCCAACCTTCAGTCCTTC |
| BoHSP90-4 | TCAAGAGCAGCCTGAAGATAAGC | TGATTTCGTCTCAGTAGCCTCCTC |
| BoHSP90-5 | TCATCCACATCATCCCCGAC | AACCAACACCAAACTGACCAATC |
| BoHSP90-6 | GTTTTACAAGAAGGCTTTCAGTCAG | GTCATCAGAGATAAACACACGCTTG |
| BoHSP90-7 | TTGTGGACTCGCCTTGCTG | TGATTTCCATCGTCTTCTTGCTC |
| BoHSP90-8 | ACTGGAAGAGTCAGAGGACGAGAAG | GTTACCAGACAGCAAGGCGAG |
| BoHSP90-9 | TGATTCTCCGTGCTGTCTTGTG | CAGACTTGTCGTTCTTGTCCACC |
| BoHSP90-10 | AGGACAGTTTGGTGTTGGGTTCTAC | AGTTCTCAGGGTCCGTTTCTTCTC |
| BoHSP90-11 | TAGTGTCCGTGTTGTTCCTTTTCTC | CTGTAACTTCGTCGGTGCTATCTTC |
| BoHSP90-12 | ACGTTACCAATCCGAAGACAAAG | CATTTGAGTCCACAACACCCTTC |

*BoActin*  ACGTGGACATCAGGAAGGAC GAACCACCGATCCAGACACT

**Table 3**: Description of motifs in Cabbage HSP90 family gene sequences

| Motif # | Sequence | width |
| --- | --- | --- |
| **1** | DSPCVLVTGKFGWSANMERJMKAQALGDSSSLEYMRGKRVLEINPDHPII | 50 |
| **2** | AEKFEFQAEVSRLLDLIINSLYSNKEIFLRELISNASDALDKIRFLSLTD | 50 |
| **3** | EVYHEWELVNEQKPIWLRNPKEVTKEEYAEFYK | 33 |
| **4** | DVNLIGQFGVGFYSAYLVADKVIVTTKHN | 29 |
| **5** | PLAVSHFTTEGEVEFKAILYVPPRAPFD | 28 |
| **6** | YNKFWENFGKNLKLGIIEDSQNRKRJAELLRFHSTKSEDEL | 41 |
| **7** | GVVDSDDLPLNVSREILQZSRI | 22 |
| **8** | YVERMKEGQKDIFYITGESKKAVENSPFLEKLIKKGYEVLYLVDPIDEYA | 50 |
| **9** | GQPELEIHIIPDKANNTJTIIDSGIGMTKEDLVBNLGTIARSGTKEFMEA | 50 |
| **10** | VTNKKTKNIKLYVRRVFISDDFDEELPEY | 29 |

|  | | | | | | | | | |
| --- | --- | --- | --- | --- | --- | --- | --- | --- | --- |
|  |  |  |  |  |  |  |  |  |  |
|  |  |  |  |  |  |  |  |  |  |
|  |  |  |  |  |  |  |  |  |  |
|  |  |  |  |  |  |  |  |  |  |
|  |  |  |  |  |  |  |  |  |  |

| **Supplementary Table 5:** Tissue-specific expression analysis of Cabbage HSP90 genes | | | | | | | | |
| --- | --- | --- | --- | --- | --- | --- | --- | --- |
| **Transcript ID** | **Bub** | **Callus** | **Flower** | **Leaf** | **Root** | **Slique** | **Stem** |  |
| BoHSP90-1 | 2.82591 | 3.49106 | 4.10647 | 4.11674 | 4.22041 | 4.34535 | 4.25 |  |
| BoHSP90-2 | 3.63647 | 4.67095 | 3.22562 | 4.42354 | 4.19548 | 3.94408 | 3.39303 |  |
| BoHSP90-3 | 7.7096 | 6.53299 | 7.89605 | 6.79258 | 5.40455 | 7.47851 | 6.64504 |  |
| BoHSP90-4 | 1.38791 | 1.69129 | -0.3012 | 3.08952 | -0.6486 | 0.29912 | 0.4228 |  |
| BoHSP90-5 | 6.50714 | 7.81837 | 9.0449 | 9.3745 | 7.96817 | 9.86103 | 9.69938 |  |
| BoHSP90-6 | -3.8069 | -0.5449 | -3.073 | -1.1824 | -3.0782 | -3.3246 | -3.3788 |  |
| BoHSP90-7 | 7.47764 | 8.06702 | 6.77681 | 7.71059 | 7.71139 | 7.44461 | 6.78182 |  |
| BoHSP90-8 | 8.55731 | 8.44705 | 8.08863 | 8.60933 | 8.51969 | 8.47405 | 8.13755 |  |
| BoHSP90-9 | 4.54965 | 6.89887 | 8.87245 | 9.31413 | 8.28627 | 9.54163 | 9.83293 |  |
| BoHSP90-10 | 2.59959 | 4.22441 | 3.04756 | 3.35851 | 1.82916 | 3.81168 | 3.08459 |  |
| BoHSP90-11 | 3.75523 | 3.80685 | 3.72746 | 4.29946 | 4.49958 | 4.65525 | 4.16265 |  |
| BoHSP90-12 | 4.78931 | 5.43045 | 2.72481 | 4.58838 | 3.04723 | 4.59292 | 3.82947 |  |
